# ncDENSE: a novel computational method based on a deep learning framework for non-coding RNAs family prediction

**DOI:** 10.1186/s12859-023-05191-6

**Published:** 2023-02-27

**Authors:** Kai Chen, Xiaodong Zhu, Jiahao Wang, Lei Hao, Zhen Liu, Yuanning Liu

**Affiliations:** 1 College of Software, Jilin University, Changchun, 130012 China; 2 Key Laboratory of Symbolic Computation and Knowledge Engineering of Ministry of Education, Jilin University, Changchun, 130012 China; 3 College of Computer Science and Technology, Jilin University, Changchun, 130012 China; 4 Graduate School of Engineering, Nagasaki Institute of Applied Science, 536 Aba-machi, Nagasaki 851-0193 Japan

**Keywords:** ncRNAs family, Dynamic Bi-GRU, DenseNet, ncDENSE

## Abstract

**Background:**

Although research on non-coding RNAs (ncRNAs) is a hot topic in life sciences, the functions of numerous ncRNAs remain unclear. In recent years, researchers have found that ncRNAs of the same family have similar functions, therefore, it is important to accurately predict ncRNAs families to identify their functions. There are several methods available to solve the prediction problem of ncRNAs family, whose main ideas can be divided into two categories, including prediction based on the secondary structure features of ncRNAs, and prediction according to sequence features of ncRNAs. The first type of prediction method requires a complicated process and has a low accuracy in obtaining the secondary structure of ncRNAs, while the second type of method has a simple prediction process and a high accuracy, but there is still room for improvement. The existing methods for ncRNAs family prediction are associated with problems such as complicated prediction processes and low accuracy, in this regard, it is necessary to propose a new method to predict the ncRNAs family more perfectly.

**Results:**

A deep learning model-based method, ncDENSE, was proposed in this study, which predicted ncRNAs families by extracting ncRNAs sequence features. The bases in ncRNAs sequences were encoded by one-hot coding and later fed into an ensemble deep learning model, which contained the dynamic bi-directional gated recurrent unit (Bi-GRU), the dense convolutional network (DenseNet), and the Attention Mechanism (AM). To be specific, dynamic Bi-GRU was used to extract contextual feature information and capture long-term dependencies of ncRNAs sequences. AM was employed to assign different weights to features extracted by Bi-GRU and focused the attention on information with greater weights. Whereas DenseNet was adopted to extract local feature information of ncRNAs sequences and classify them by the full connection layer. According to our results, the ncDENSE method improved the Accuracy, Sensitivity, Precision, F-score, and MCC by 2.08$\%$, 2.33$\%$, 2.14$\%$, 2.16$\%$, and 2.39$\%$, respectively, compared with the suboptimal method.

**Conclusions:**

Overall, the ncDENSE method proposed in this paper extracts sequence features of ncRNAs by dynamic Bi-GRU and DenseNet and improves the accuracy in predicting ncRNAs family and other data.

## Background

RNAs in organisms can be classified into two main categories, namely, coding RNAs [1] and non-coding RNAs (ncRNAs) [2]. A major difference between these two types of RNAs lies in their protein encoding ability [3]. For instance, messenger RNA (mRNA) in coding RNA [4] can form proteins that are essential for life activities by means of transcription and translation [5]. Although ncRNAs are not directly involved in the formation of encoded proteins, they can guide protein synthesis and indirectly participate in the translation and transcription processes [6]. As human genome annotation has become more sophisticated, researchers have found that only 1-2$\%$ of all human genes can directly encode proteins [7], however, it is found that at least 76$\%$ of the genome has the ability to produce transcription products, consequently, a large number of unknown ncRNAs play essential roles in life activities [8]. ncRNAs can be classified according to their structure, function, and coding gene location. The current research hotspots of ncRNAs include microRNAs (miRNAs), long-stranded non-coding RNAs (lncRNAs), ribosomal RNAs (rRNAs) and tRNAs [9]. Of them, miRNAs are the nucleotide sequences approximately 22 nt in length, which can reduce the expression of mRNA by base complementary pairing at the transcriptional level [10]. lncRNAs are ncRNAs greater than 200 nt in length, which are the important component of the non-coding genome. Typically, lncRNAs function to regulate transcribed RNAs and protein translation, and are involved in various regulatory physiological and pathological processes [11]. rRNAs, which are essential for life, play a regulatory role in cells and participate indirectly in protein transcription [12]. Other famous ncRNAs include ribozymes [13] and Intron$\_$RNA [14]. Internal ribosome entry sites (IRES [15]), leader [16] and riboswitch [17] belong to mRNA fragments. Notably, ribozymes belong to a class of catalytically active RNA molecules exiting in nature, which can catalyze reactions such as RNA breakage and ligation. Intron$\_$RNA is the RNA transcribed from intron genes, after being transcribed into RNA, it can interact with many substances to help link exons together in the correct order. IRES, as an effective translation initiator, is commonly constructed in the middle of ribosome and mRNA. Leader is the upstream segment of the start codon in mRNA, which can regulate mRNA transcription. As for riboswitch, it is a regulatory segment of mRNA, which plays a role in regulating gene expression in prokaryotes.

**Fig. 1:**
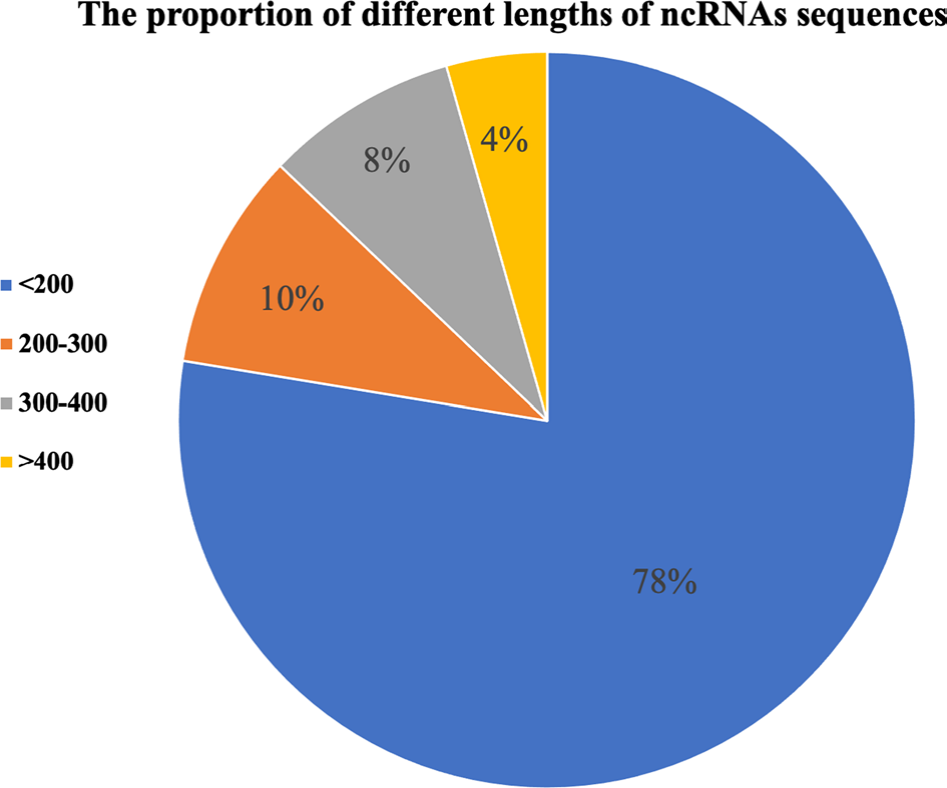
The proportion of different lengths of ncRNAs sequences

With the rapid development of high-throughput technology, an increasing number of unknown ncRNAs have been discovered. ncRNAs exert indispensable functions in life activities, so it is important to study the functions of these unknown ncRNAs. Plenty of evidence shows that ncRNAs of the same family have similar functions, therefore, accurately predicting the belonging families of unknown ncRNAs can initially predict the functions of unknown ncRNAs. The traditional method to predict the belonging families of unknown ncRNAs is the bioassay method, but it is labor-intensive and cannot address the large amount of data generated by high-throughput technologies [18]. Therefore, convenient and fast computational methods are applied to predict the ncRNAs families. Computational methods for ncRNAs family prediction can be divided into two types, the first type is to predict based on secondary structure features of ncRNAs, and the second one is to predict according to sequence features of ncRNAs. In the first type, the main workflow is to first predict the secondary structure of ncRNAs, and later predict ncRNAs families based on the secondary structure features of ncRNAs. However, due to the inaccurate ncRNAs secondary structure prediction, there is a lot of useless information in the extracted secondary structure features of ncRNAs, which affects the performance of ncRNA family prediction. The prediction methods based on ncRNAs secondary structure features include GraPPLE [19], RNAcon [20], and nRC [21]. Among them, the GraPPLE method extracts the secondary structure graph information of ncRNAs using summary statistics with local–global properties, and the extracted information is classified by the SVM method afterwards. As for RNAcon, it first distinguishes coding RNAs from ncRNAs by the SVM-based TNC method, and then classifies different ncRNA classes by the RandomForest classifier based on the graph information regarding the secondary structure of ncRNAs. Moreover, the nRC method utilizes the IPknot algorithm to predict RNA secondary structure with pseudoknots, then uses the MoSS decision tree pruning algorithm to obtain substructure, and finally adopts the convolutional neural network as a classifier. The main workflow of the second type of prediction is to directly extract the sequence features of ncRNAs and then perform ncRNAs prediction based on the sequence features of ncRNAs, the main methods are ncRFP [22], ncDLRES [23] and the method proposed by T.M.R. Noviello, F. Ceccarelli et al [24]. With regard to ncRFP, it extracts the ncRNAs sequence features by the RNN model and then classifies them by a fully connected layer. ncRFP simplifies the prediction process and improves the prediction accuracy, however, due to the different lengths of ncRNAs sequences, they are padded or segmented to the same sequence length before LSTM is used to extract features, which not only loses a lot of ncRNAs sequence feature information, but also adds a lot of useless information. ncDLRES makes full use of the ncRNAs sequence features by using dynamic LSTM model, but there are still problems such as low accuracy and low efficiency. In this regard, there is still much room for improvement in ncDLRES performance.The optimal method proposed by T.M.R. Noviello, F. Ceccarelli, M. Ceccarelli et al used 1k-mer method to encode the sequences of ncRNAs, and the encoded ncRNAs sequences were input to a improved CNN model to extract the local features of ncRNAs, which achieved good results, but the method was only applicable to ncRNAs with sequence less than 200 nucleotides. As shown in Fig. 1, the database used in this paper contains ncRNAs with sequences larger than 200 nucleotides, so the ncDENSE method can be used in a broader range.

**Fig. 2:**
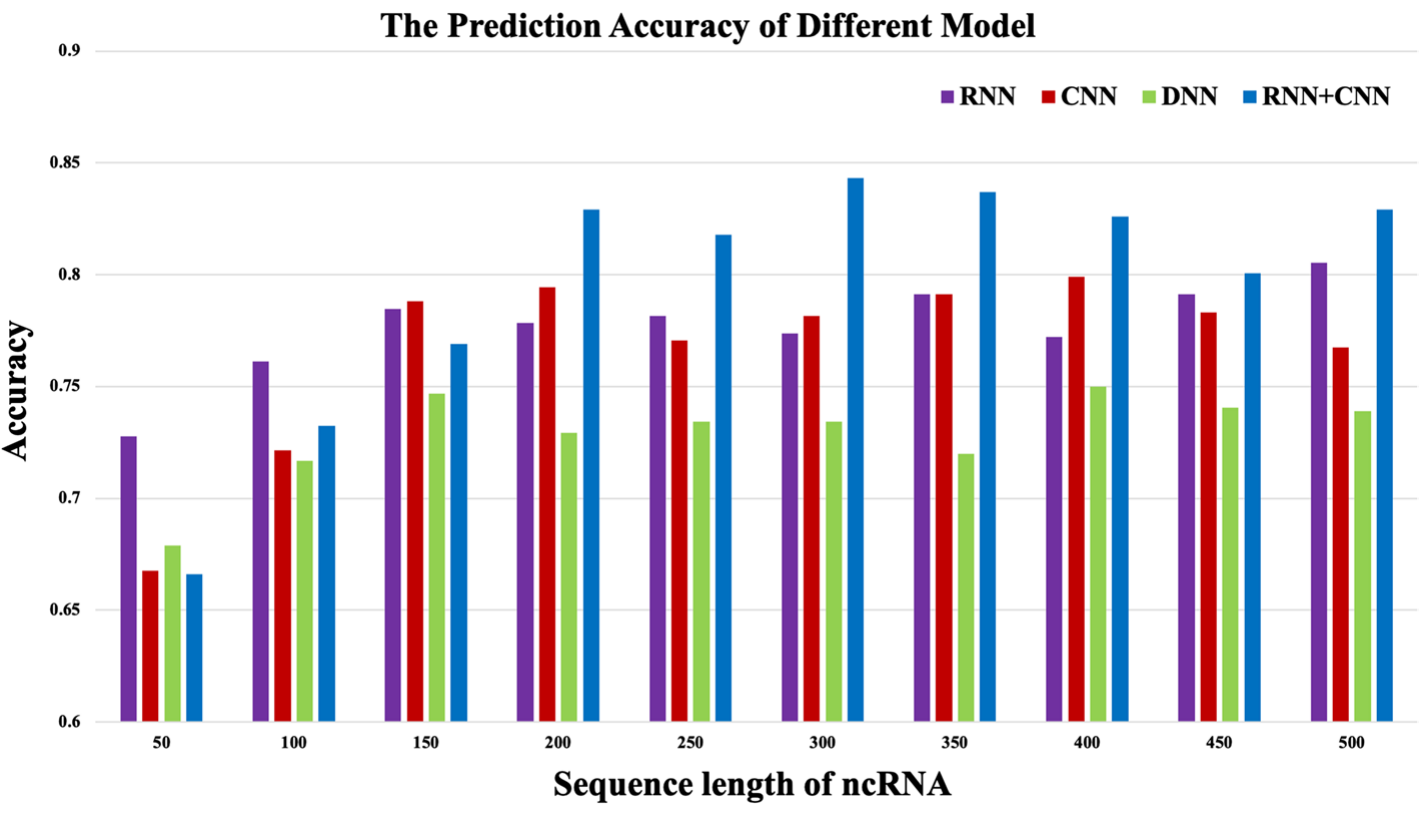
Comparison of the accuracy of different deep learning models on different sequence lengths of ncRNAs

In this paper, four representative deep learning models, including deep neural networks (DNN), convolutional neural networks (CNN), recurrent neural networks (RNN), and the combination of recurrent and convolutional neural networks (RNN+CNN), were compared. Data in Fig. 2 indicated that the RNN+CNN model was generally superior to the other three models for different lengths of ncRNAs sequences, which was thereby selected for ncRNAs family prediction. The model included the dynamic Bi-GRU, the Attention Mechanism, and the DenseNet, which not only simplified the prediction process but also improved the accuracy compared with the existing methods.

## Result

ncRNAs include primary structure, secondary structure, and tertiary structure. Of them, the primary structure corresponds to the sequence structure of ncRNAs, while the secondary structure is associated with the planar structure of ncRNAs, and the tertiary structure is related to the spatial structure of ncRNAs [25]. Typically, the primary structure of ncRNAs can be accurately obtained by simple biological experimental methods. Meanwhile, the secondary structure of ncRNAs, which a two-dimensional structure formed by bases in the sequence matching each other through hydrogen bonds, is divided into various forms, including helices and single-stranded. The single-stranded from is further divided into hairpin loop, inner loop, synaptic loop, and multi-branching structure, due to the diversity of secondary structure of ncRNAs, as a result, it is a complex process with a low accuracy in obtaining the secondary structure of ncRNAs. The tertiary structure of ncRNAs is a three-dimensional spatial structure formed by further folding of secondary structure stems, loops, and pseudoknots and other modules. Classical methods for predicting the tertiary structure of ncRNAs are knowledge-based and physical methods, however, due to the instability and easy change by external environmental influences, it is more difficult to directly obtain the tertiary structure of ncRNAs. Sequence structure, planar structure, and spatial structure of ncRNAs contain features of the ncRNAs family, and they can be used as input to the RNN+CNN ensemble deep learning model. The primary structure of ncRNAs has family consistent sequence fragments, therefore, deep learning models can be used to extract ncRNAs sequence features, which can later be used to classify the belonging family of unknown ncRNAs mainly by ncRFP and ncDLRES. Compared with ncRFP, the method proposed in this paper dynamically input the base sequences of ncRNAs into the dynamic Bi-GRU model after encoding, which thus avoided cutting and padding ncRNAs sequences and retaining the full feature information of ncRNAs sequences. Relative to ncDLRES, ncDENSE utilizes Bi-GRU [26, 27] instead of LSTM, which has three gate information while GRU only retains two gate information, thereby significantly improving the code running efficiency. Bi-GRU belongs to the bidirectional RNN model, which not only retains the base information before the current base of ncRNAs sequence, but also records the base information after the current base, contributing to better extracting ncRNAs sequence features. In the ncDLRES method, ResNet [28] is employed to extract the local feature information of ncRNA sequences, and the DenseNet [29] model used in this paper better solved the problem of gradient disappearance when the deep network was back propagated. Compared with the ncRNAs family prediction method by ncRNAs secondary structure, the method proposed in this paper directly skipped the prediction of ncRNAs secondary structure, which not only improved the prediction accuracy but also enhanced the prediction efficiency.

Compared with the previously proposed method, the ncDENSE method was significantly improved in result data in two aspects. The first improvement was reflected in the overall data by comparing data of each method through the average of ten-fold cross-validation, while the second improvement was reflected in the individual ncRNA familiy by comparing the performance of 13 ncRNAs families predicted by different methods.

### Comparison of overall data

**Fig. 3:**
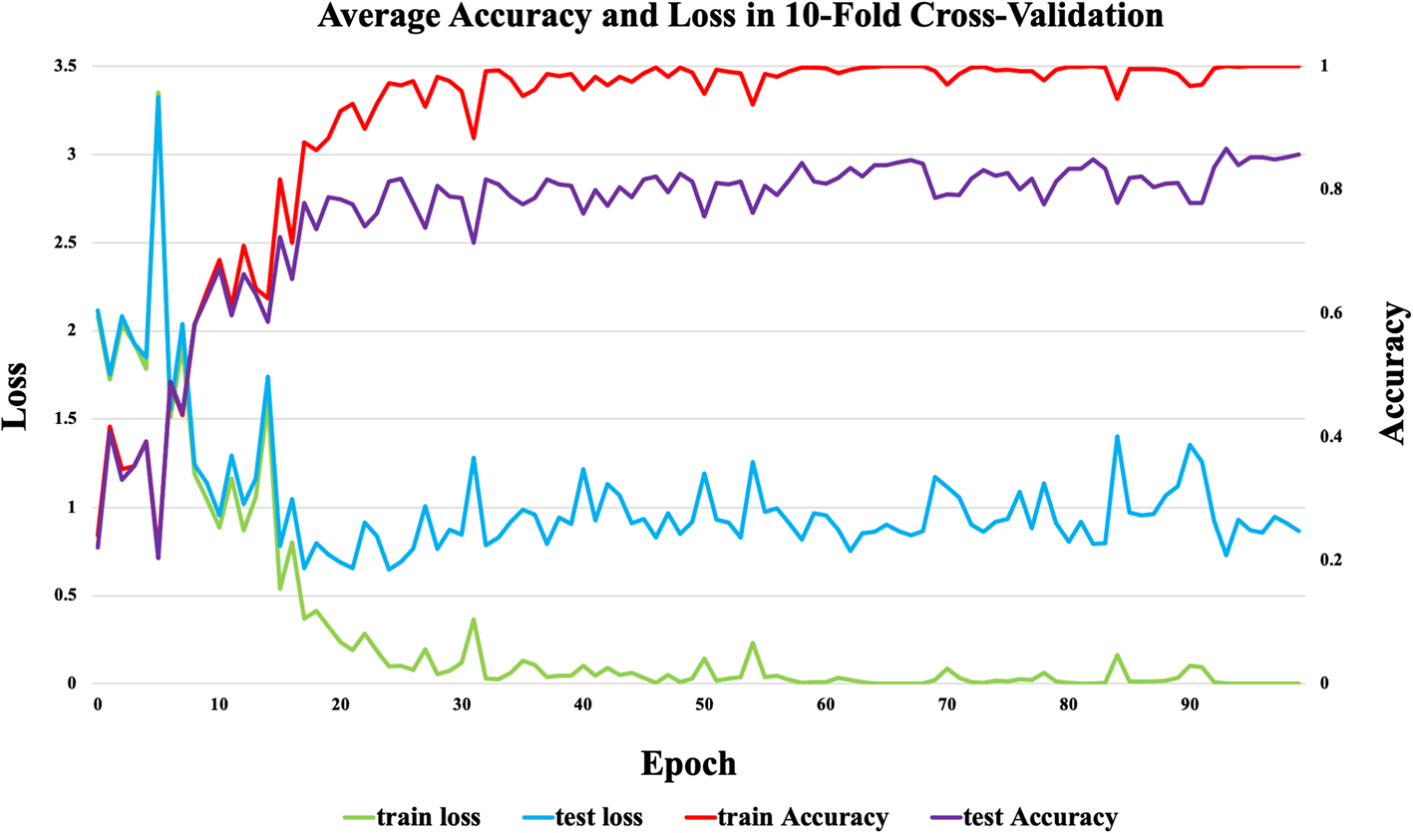
Accuracy and loss rate of 10-fold cross-validation

In the model training process, ten-fold cross-validation was adopted, in which all ncRNAs data were divided into ten parts, with nine of them being used as the training set while the remaining one as the test set in turn, and each model training consisted of 100 epochs. Figure 3 shows the accuracy and loss rate of ncDENSE in the 10th fold cross-validation. As observed, although the curves fluctuated, there was no overfitting or underfitting. The highest model accuracy and lowest loss rate in training were obtained at the epochs of 92. To make the proposed method more convincing, the Accuracy, Sensitivity, Precision, F-score, and MCC of six ncRNAs family prediction methods, namely GeaPPLE, ncRFP, nRC, RNAcon, ncDLRES, and ncDENSE, were compared. Accuracy represented the percentage of correctly predicted ncRNAs among all ncRNAs, Sensitivity indicated the percentage of correctly predicted ncRNAs in each ncRNA family, Precision was the percentage of correctly predicted data for each family, F-score was defined as the weighted average of Sensitivity and Precision, and MCC was the metric used to measure classification performance. Accuracy, Sensitivity, Precision, F-score, and MCC were calculated as follows(Eqs, 1 -  5), with TP, TN, FP, and FN representing True Positives, True Negatives, False Positives, and False Negatives, separately.

1 $$\begin{aligned}{} & {} { Accuracy }=\frac{T P+T N}{T P+T N+F P+F N} \end{aligned}$$

2 $$\begin{aligned}{} & {} { Sensitivity }=\frac{T P}{T P + F N} \end{aligned}$$

3 $$\begin{aligned}{} & {} { Precision }=\frac{T P}{T P + F P} \end{aligned}$$

4 $$\begin{aligned}{} & {} { F-score }=\frac{2 \times T P}{2 \times T P + F P + F N} \end{aligned}$$

5 $$\begin{aligned}{} & {} \textrm{MCC}=\frac{T P \times T N-F P \times F N}{\sqrt{(T P+F P) \times (T P+F N) \times (F P+T N) \times (F N+T N)}} \end{aligned}$$

Table 1 displays the mean values of the six ncRNAs family prediction methods obtained on the test set by ten-fold cross-validation. In this paper, the proposed ncDENSE method had the best performance in all performance metrics and improved the Accuracy, Sensitivity, Precision, F-score, and MCC by 2.08$\%$, 2.33$\%$, 2.14$\%$, 2.16$\%$, and 2.39$\%$, respectively, compared with the suboptimal values.

**Table 1:** Comparison of the performance of multiple methods

Method	Accuracy	Sensitivity	Precision	F-score	MCC
RNAcon	0.3737	0.3787	0.4500	0.3605	0.3341
GeaPPLE	0.6487	0.6684	0.7325	0.7050	0.6857
nRC	0.6960	0.6889	0.6878	0.6878	0.6627
ncRFP	0.7972	0.7878	0.7904	0.7883	0.7714
ncDLRES	0.8430	0.8344	0.8419	0.8407	0.8335
ncDENSE	0.8687	0.8677	0.8703	0.8667	0.8574

### Detailed data comparison

**Fig. 4:**
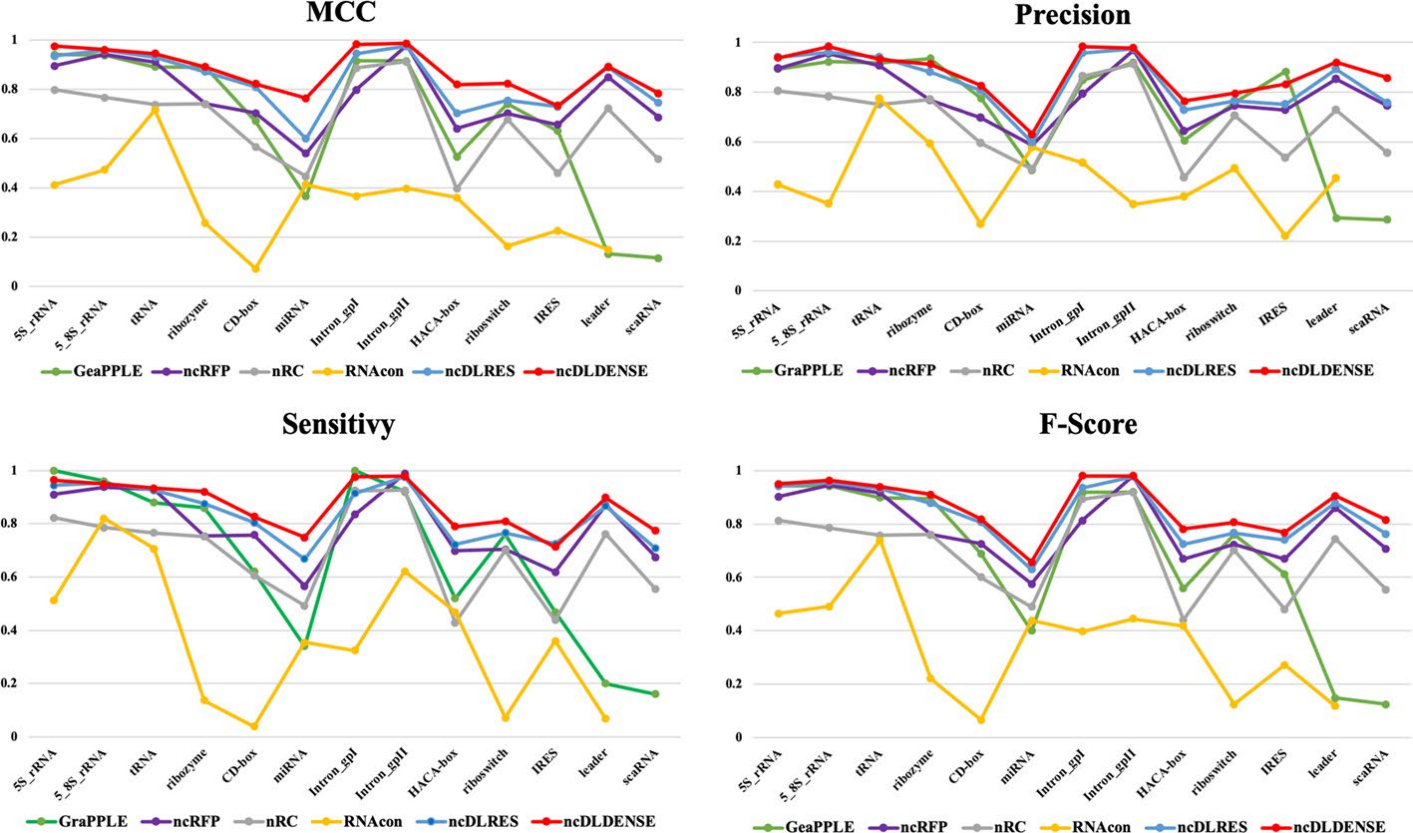
Performance comparison of ncRNAs family prediction on different methods

**Fig. 5:**
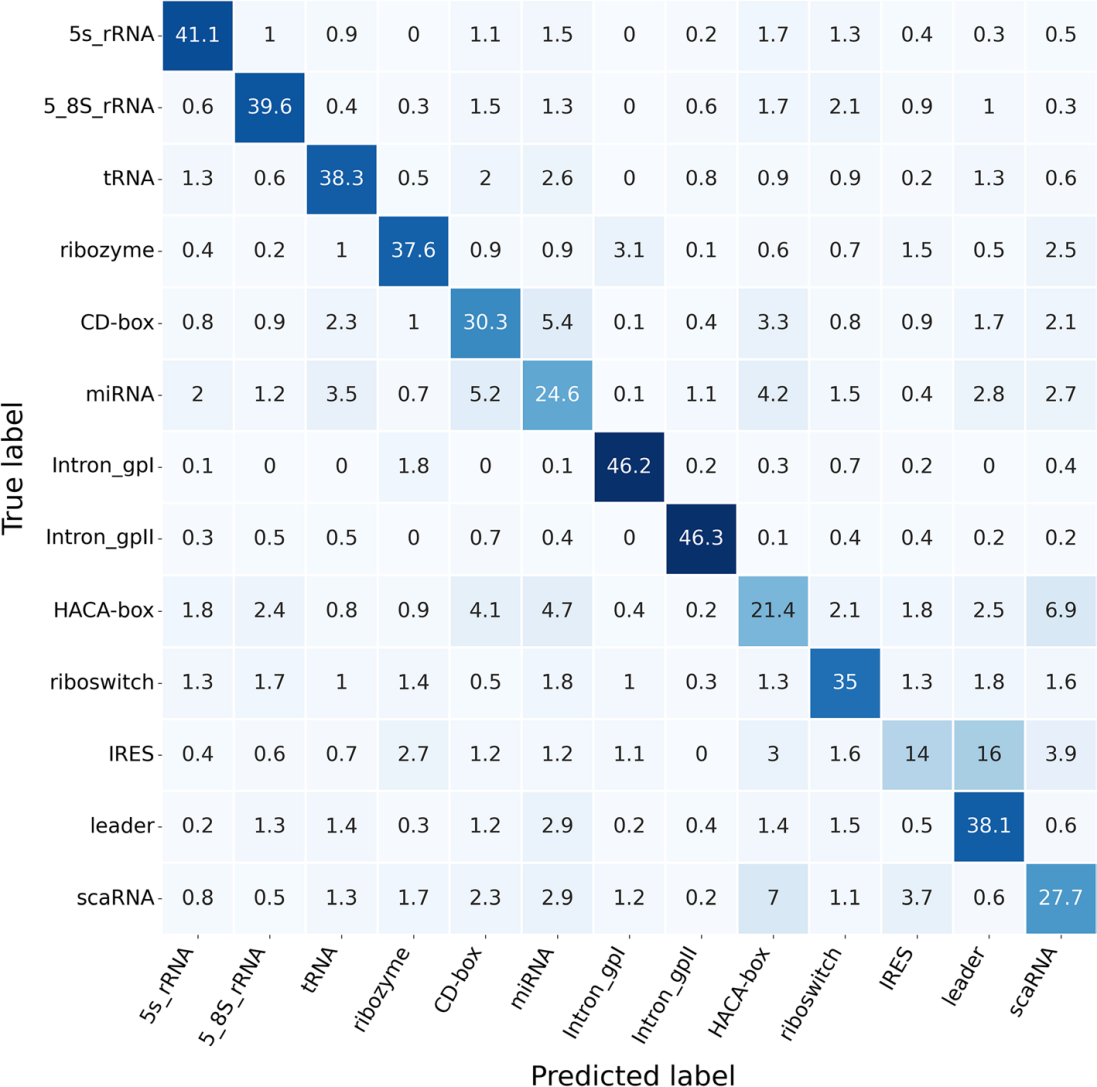
Detailed data of the nRC method for predicting ncRNAs family

**Fig. 6:**
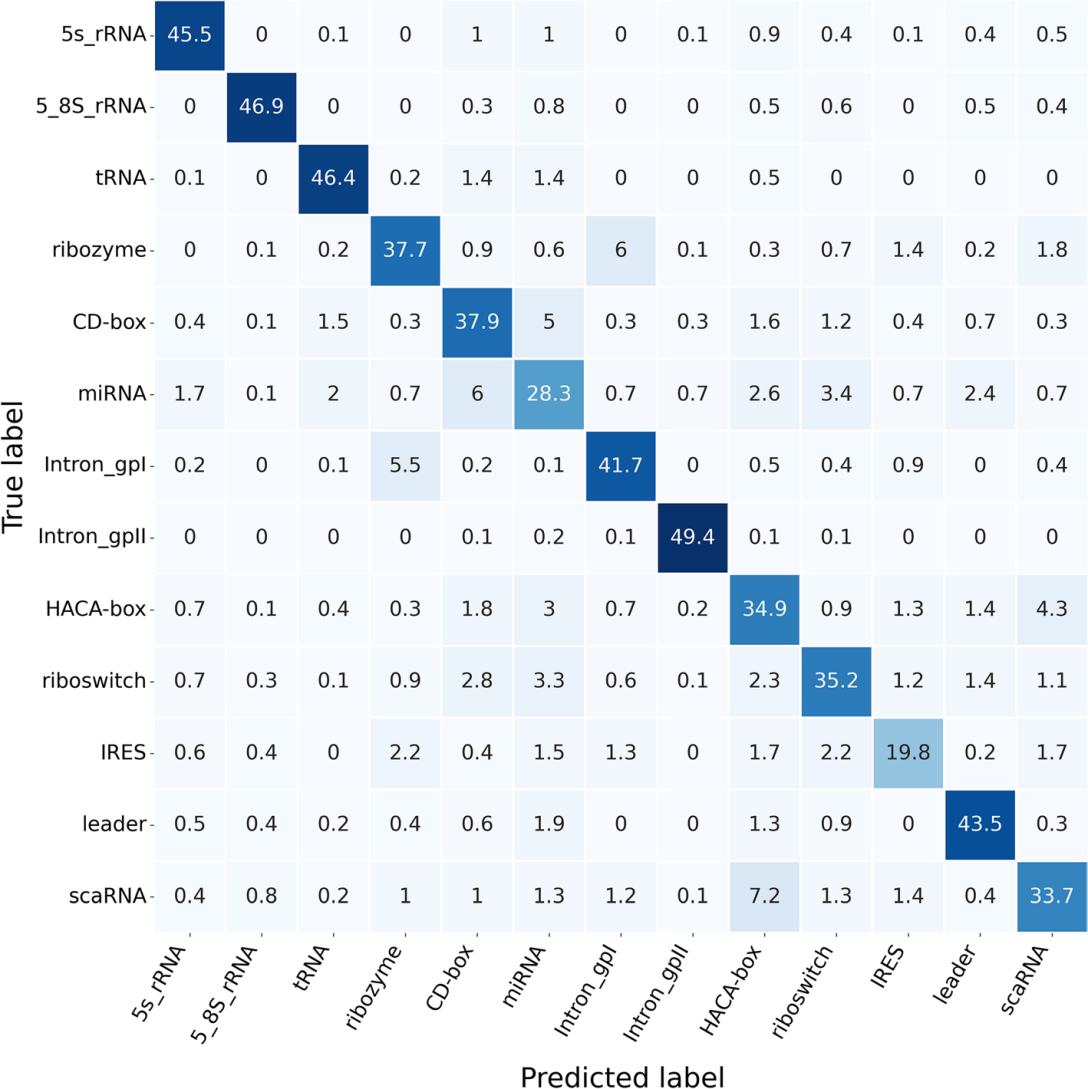
Detailed data of the ncRFP method for predicting ncRNAs family

**Fig. 7:**
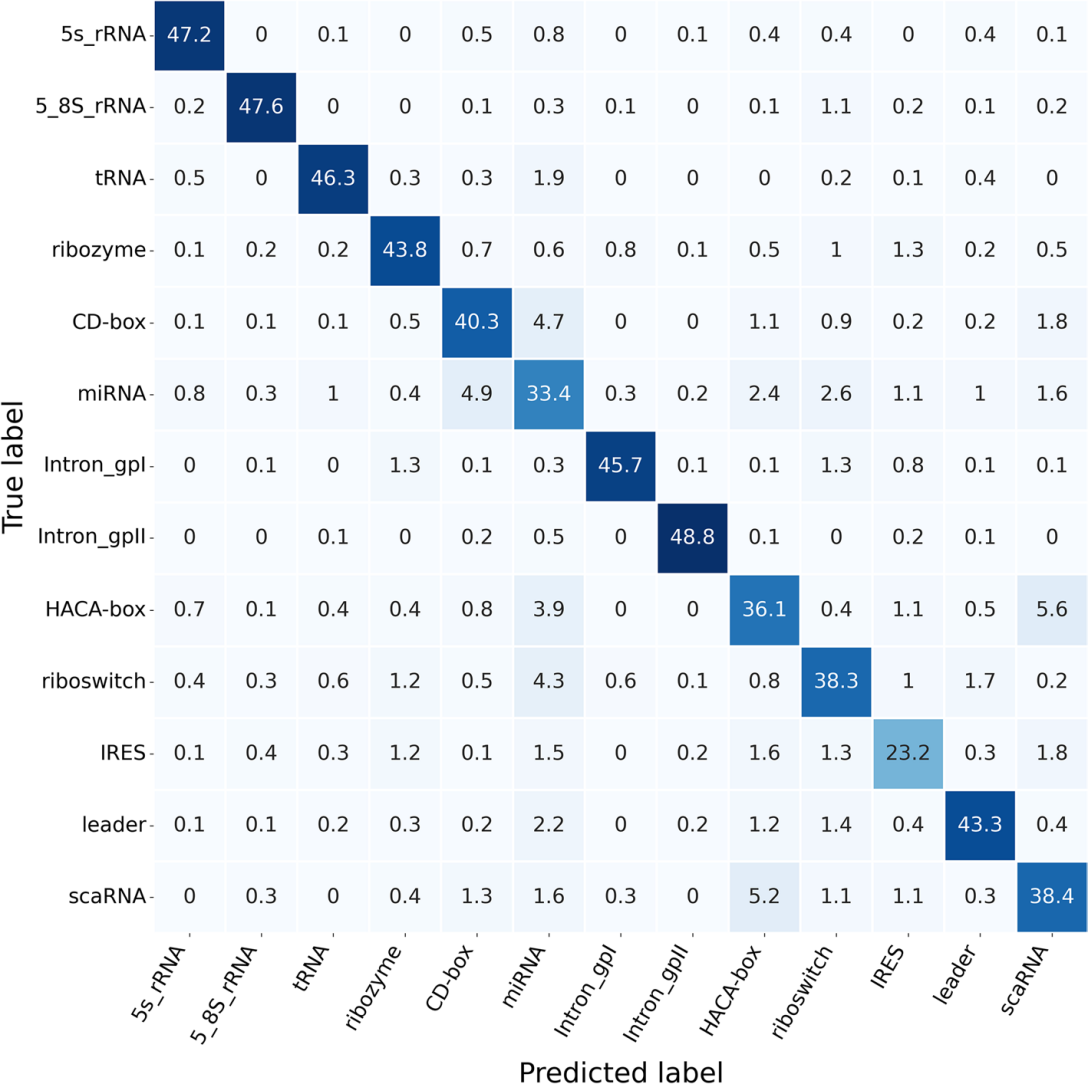
Detailed data of the ncDLRES method for predicting ncRNAs family

**Fig. 8:**
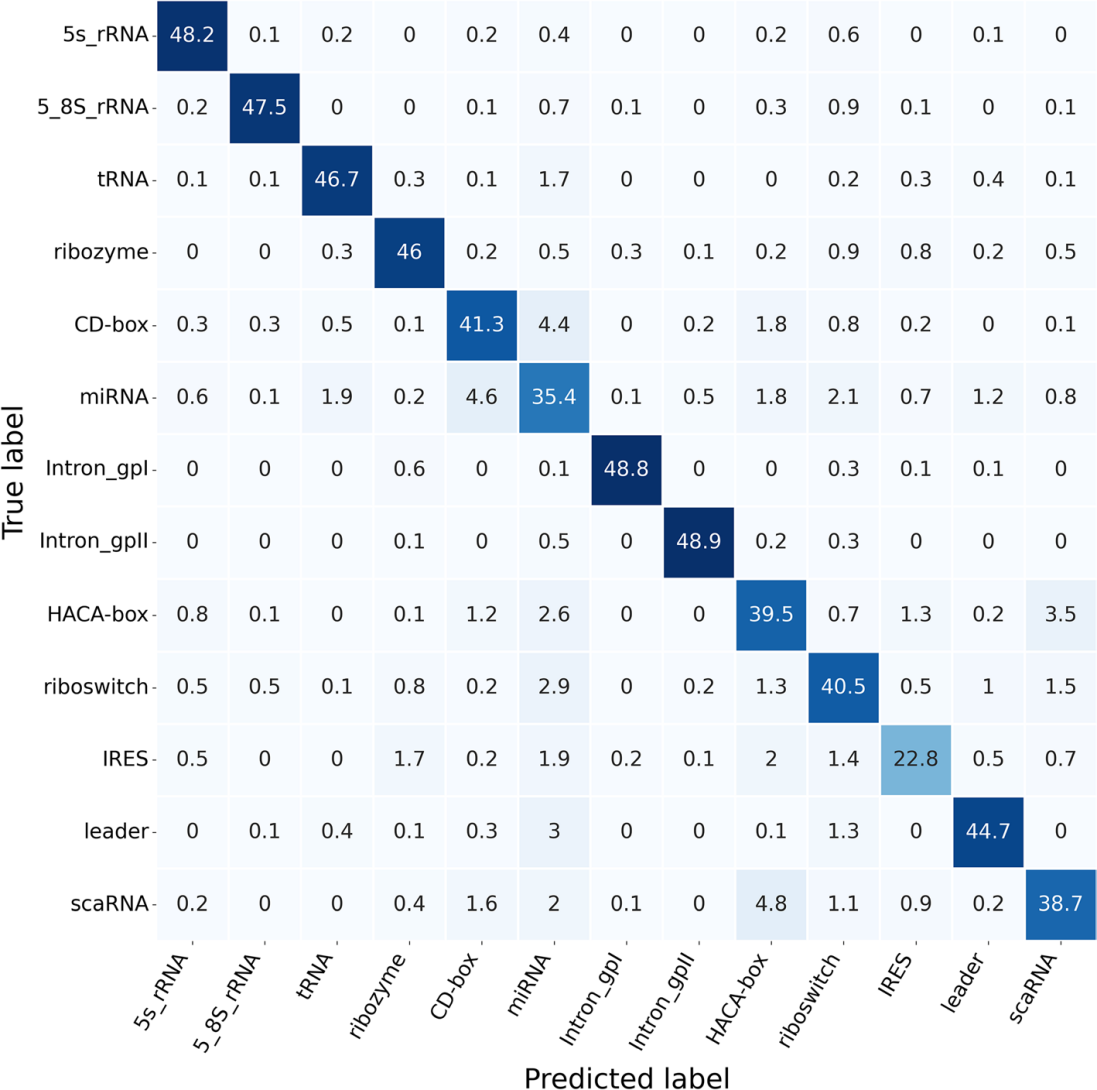
Detailed data of the ncDENSE method for predicting ncRNAs family

This section focused on comprehensively comparing ncRNAs family predictions. To achieve this end, ncRNAs data were divided into 13 categories according to ncRNAs families, including microRNAs, 5 S$\_$rRNA, 5.8 S$\_$rRNA, ribozymes, CD-box, HACA-box, scaRNA, tRNA, Intron$\_$gpI, Intron$\_$gpII, IRES, leader and riboswitch. Figure 4 exhibits the Sensitivity, Precision, F-score, and MCC data calculated for each ncRNAs family in the six ncRNAs family prediction methods, including GeaPPLE, ncRFP, nRC, RNAcon, ncDLRES, and ncDENSE. As observed from data in Fig. 4, compared with the other five methods, the ncDENSE method achieved great improvements in MCC, Precision, Sensitivity, and F-Score for the prediction of each ncRNAs family, even though it did not well predict a few ncRNAs families. Figures 5, 6, 7 and 8 present more details of each ncRNA family predicted by the four methods, namely, ncDENSE, ncDLRES, ncRFP, and nRC, in the ten-fold cross-validation. To facilitate observation and comparison, the heat map method was used to record the data in this paper, and the data inside each heat map represents the average of the predicted ncRNAs results of this method by ten-fold cross-validation. The horizontal coordinates represent the predicted results of ncRNAs, and the vertical coordinates represent the true classification of ncRNAs. For example, the row data labeled 5s_rRNA in Figure 4 indicates that 48.2 5s_rRNAs were successfully predicted as 5s_rRNA, 0.1 5s_rRNA was predicted as 5_8s_rRNA, 0.2 5s_rRNA was predicted as tRNA, no 5s_rRNA was predicted as ribozyme, and so on. As discovered from Figs. 5, 6, 7 and 8, the ncDENSE method only predicted two ncRNAs families, 5.8S_rRNA and IRES, with fewer correct predictions of ncRNA family than the ncDLRES method. The number of correctly predicted ncRNAs was improved in other ncRNA families.

### Time efficiency comparison

**Fig. 9:**
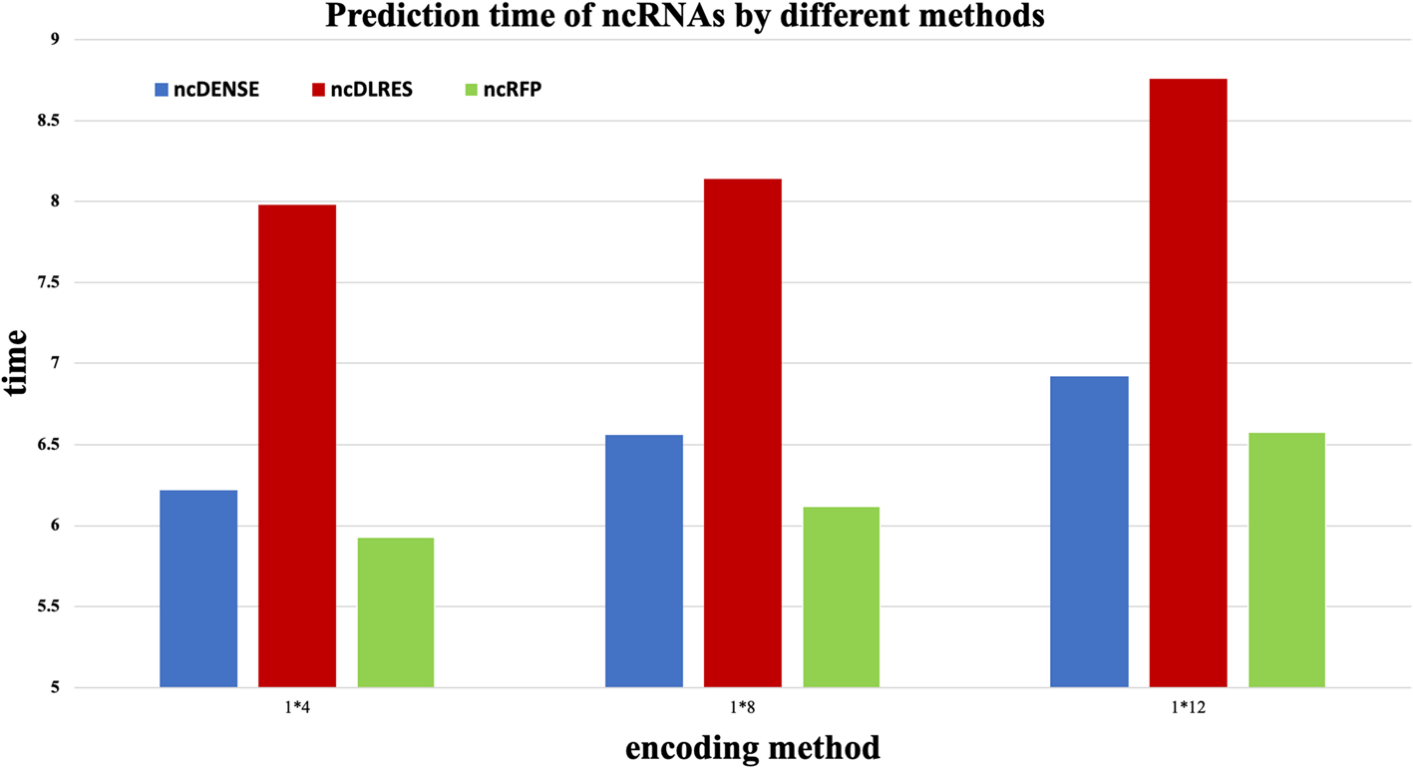
Prediction time of ncRNAs by different methods

This section compares the time efficiency of three ncRNAs prediction methods: ncRFP, ncDLRES, and ncDENSE. ncRFP, ncDLRES, and ncDENSE methods all perform classification prediction of ncRNAs by extracting the sequence features of ncRNAs, because these three methods skip the process of predicting the secondary structure of ncRNAs, so time efficiency of these three methods is much greater than that of the methods that perform classification prediction of ncRNAs by secondary structure features of ncRNAs. ncRFP method uses a model of bidirectional LSTM and full linkage layer, ncDLRES uses a model of LSTM and ResNet, and ncDENSE uses a dynamic Bi-GRU and attention mechanism and DenseNet model. Because LSTM uses three gates to extract features from sequence data while GRU uses only two gates to extract features from sequence data, the operational efficiency of GRU is greater than that of LSTM, and therefore the operational efficiency of the ncDENSE method is greater than that of the ncDLRES method. ncDENSE and ncDLRES methods both use an improved convolutional model to extract local features of ncRNAs sequences, and ncRFP only uses the full concatenation layer for classification, so the ncRFP method runs more efficiently than the ncDENSE method and ncDLRES method. As shown in Figure 9, the running time of ncRFP method is smaller than that of ncDENSE method and ncDLRES method. ncDENSE method is larger than ncDLRES method in terms of running efficiency and accuracy. Although the running efficiency of ncRFP is greater than that of the ncDENSE method, the accuracy of the ncDENSE method is much greater than that of the ncRFP method. We tested the running time of 1264 ncRNAs predictions using a 3090ti graphics card. ncRFP method was only 0.5 s faster than ncDENSE method, but the accuracy of ncDENSE method was 7.15$\%$ higher than that of ncRFP method. Therefore, by comparing the time efficiency and accuracy of the three methods, the ncDENSE method was superior to the ncRFP method and the ncDLRES method.

## Discussion

RNAs are the very important biological macromolecules, which play an indispensable role in life activities. RNAs can be divided into two categories based on their protein encoding ability, namely, coding RNAs that can directly participate in protein transcription and translation, and ncRNAs that can indirectly participate in protein translation and transcription. Notably, ncRNAs are associated with structural complexity and functional diversity, which have posed a major obstacle for researchers to unravel their mysteries. In recent years, biologists have made unremitting efforts to find that ncRNAs of the same family have similar functions. Therefore, by identifying the belonging families of unknown ncRNAs, it is possible to initially determine their functions and thus promote the study on ncRNAs. The existing methods used to identify ncRNAs are divided into two categories, namely, biological experiment-based and computational-based methods. Although biological experiment-based methods can achieve high accuracy in some fields, they are labor-intensive and cannot meet the demand for high throughput. Meanwhile, computational-based methods can be further subdivided into two categories, including secondary structure-based and sequence-based characterization of ncRNAs. To be specific, secondary structure-based ncRNAs family prediction methods include RNAcon, GeaPPle, and nRC, but these methods are inefficient because the acquisition of ncRNAs secondary structure involves a complex process with a low accuracy rate. Other methods adopted to classify ncRNAs based on their sequence characteristics are ncRFP and ncDLRES. Of them, the ncRFP method can predict ncRNAs families by sequence features of ncRNAs, which simplifies the prediction process while improving the prediction accuracy compared with RNAcon, GeaPPle, nRC, and other methods. However, this method uses the static LSTM method to fill or intercept the unequal ncRNAs sequences, which may thus miss the characteristic information of some ncRNAs and add some useless characteristic information, thereby decreasing the ncRNAs family prediction accuracy. By contrast, ncDLRES used dynamic LSTM to avoid the problem of missing ncRNAs sequence feature information, nonetheless, it also adopts a one-way RNN model, which can only extract base information before the current base, to extract ncRNAs sequence contextual feature information, resulting in inadequate feature extraction. In this paper, a novel ncDENSE method integrating dynamic BiGRU + Attention Mechanism + DenseNet model was proposed. On the one hand, the dynamic Bi-GRU is improved by not filling or cutting ncRNAs sequences, so that the sequence features of ncRNAs can all be input into the Bi-GRU model. On the other hand, Bi-GRU is a bidirectional RNN model, which retains the base information before the current base and records the base information after the current base, so that the contextual feature information of ncRNAs sequences can be fully extracted. Besides, the Attention Mechanism can assign more weights to the important features of ncRNAs sequences so that attention can be subtly and rationally adjusted to shift, thus ignoring irrelevant information and amplifying important information [30]. Further, DenseNet uses dense connectivity to better solve the problem of back propagation gradient disappearance in deep networks.

Compared with the five previously proposed methods, our proposed ncDENSE method showed great improvements in both the overall data of ncRNAs and the data of individual ncRNAs families. Table 1 displays the mean values of the six methods obtained by ten-fold cross-validation. Clearly, the ncDENSE method improved the Sensitivity, Precision, F-score, and MCC by 3.33$\%$, 2.12$\%$, 2.6$\%$, and 2.39$\%$, respectively, compared with the suboptimal method. Besides, its Accuracy was improved by 2.57$\%$ relative to the next best method. Figure 4 displays data comparison results of 13 ncRNAs families between ncDENSE and ncDLRES methods. Apparently, the ncDENSE method outperformed ncDLRES in terms of Sensitivity, Precision, F-score, and MCC on nine ncRNAs families, namely, microRNAs, ribozymes, CD-box, HACA-box, scaRNA, Intron_gpI, Intron_gpII, IRES, leader, and riboswitch. In the 5S_rRNA and tRNA families, the ncDENSE method was only slightly inferior to the ncDLRES method in terms of Precision data; while in the IRES family, only the Sensitivity data obtained by the ncDENSE method were inferior to those of the ncDLRES method. In Figs. 5, 6, 7 and 8, it was found that the nRC method predicted the least number of correct ncRNAs family. All the methods used in Figures  5, 6, 7 and  8 predicted ncRNAs families by the ncRNA sequence features. The numbers of correctly predicted ncRNAs for the ncDENSE method (Fig. 8), ncDLRES method (Fig. 7), and ncRFP method (Fig. 6) were 5490, 5324, and 5009, respectively. Compared with the ncDLRES method, the ncDENSE method did not performed well in predicting 5.8S_RNA and IRES families. Moreover, compared with the ncRFP method, the ncDENSE method does not well predicted the Intron_II family. Therefore, on the whole, the ncDENSE method had significantly enhanced performance in predicting ncRNAs family compared with the other five methods.

## Conclusion

The prediction of ncRNAs family can initially determine the functions of ncRNAs. In the face of massive high-throughput ncRNA sequence data, biological experiment-based methods cannot meet the demands for prediction, while the existing main computational methods are associated with the problem of complicated processes and low accuracy. In this paper, a novel computational method for ncRNAs family prediction, ncDENSE, is proposed. In summary, ncDENSE displays four advantages.

First: The ncDENSE method predicts ncRNAs families by extracting sequence features of ncRNAs, which skips the process of obtaining the secondary structure of ncRNAs, improves the ncRNAs family prediction accuracy and simplifies the prediction process.

Second: The ncDENSE method abandons the traditional padding and segmentation of ncRNAs sequences, as a result, the full features of ncRNAs sequences are fully fed into the deep learning model.

Third: The ncDENSE method adopts a bidirectional RNN model-Bi-GRU, which, compared with the unidirectional RNN model, can retains the base information before the current base and records the base information after the current base information, thus facilitating the extraction of ncRNAs sequence contextual features.

Fourth: The ncDENSE method utilizes the DenseNet network to extract local features of ncRNAs sequences, which helps to better mitigate the gradient disappearance of back propagation in the deep networks by dense connectivity.

As for the two ncRNAs families, 5S_RNA and IRES, the ncDLRES method predicts slightly better results than the ncDENSE method. Consequently, the ncDENSE method can be integrated with the ncDLRES method to build the ncRNAs family identification website to provide better help for researchers.

## Methods

### Data collection and processing

**Fig. 10:**
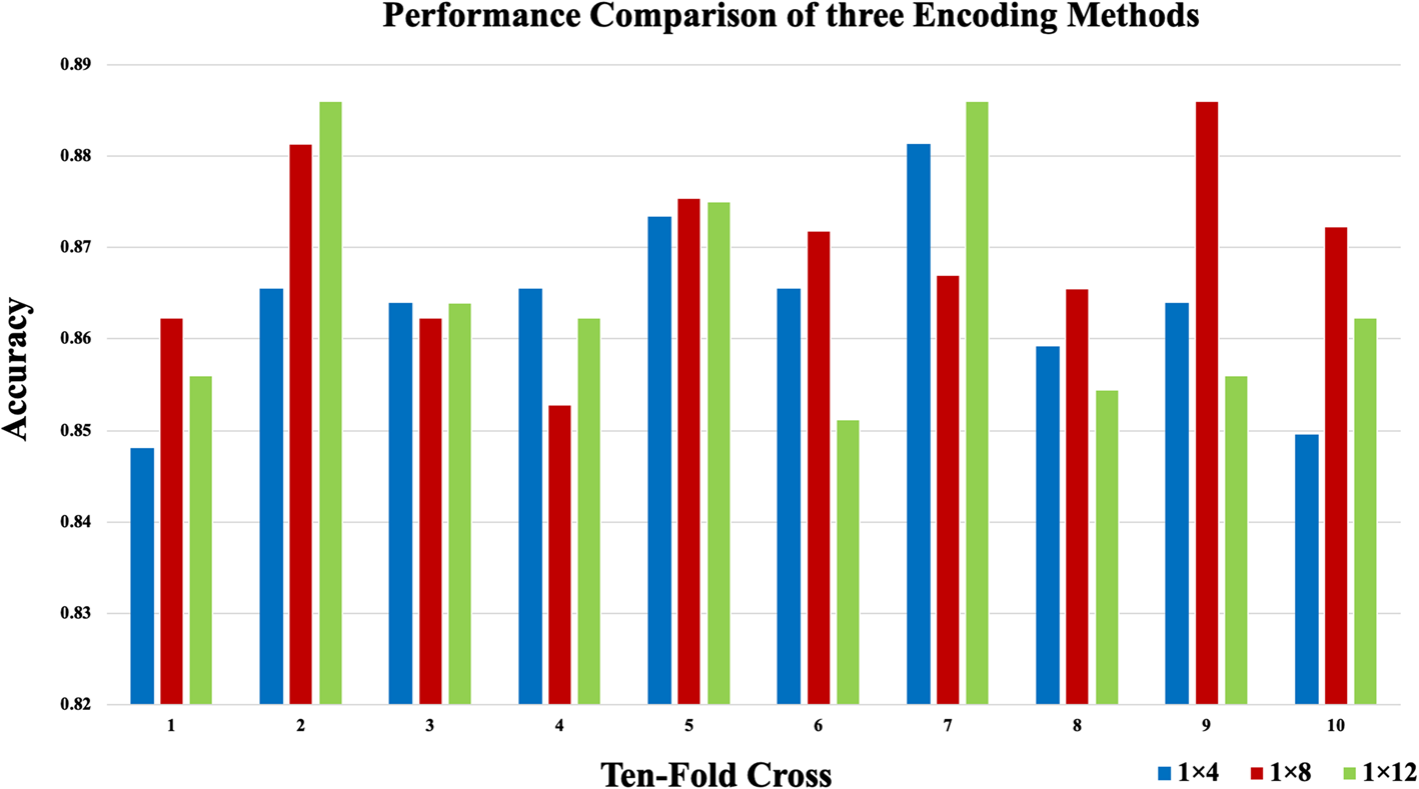
Accuracy of ten-fold cross-validation with different coding methods

**Fig. 11:**
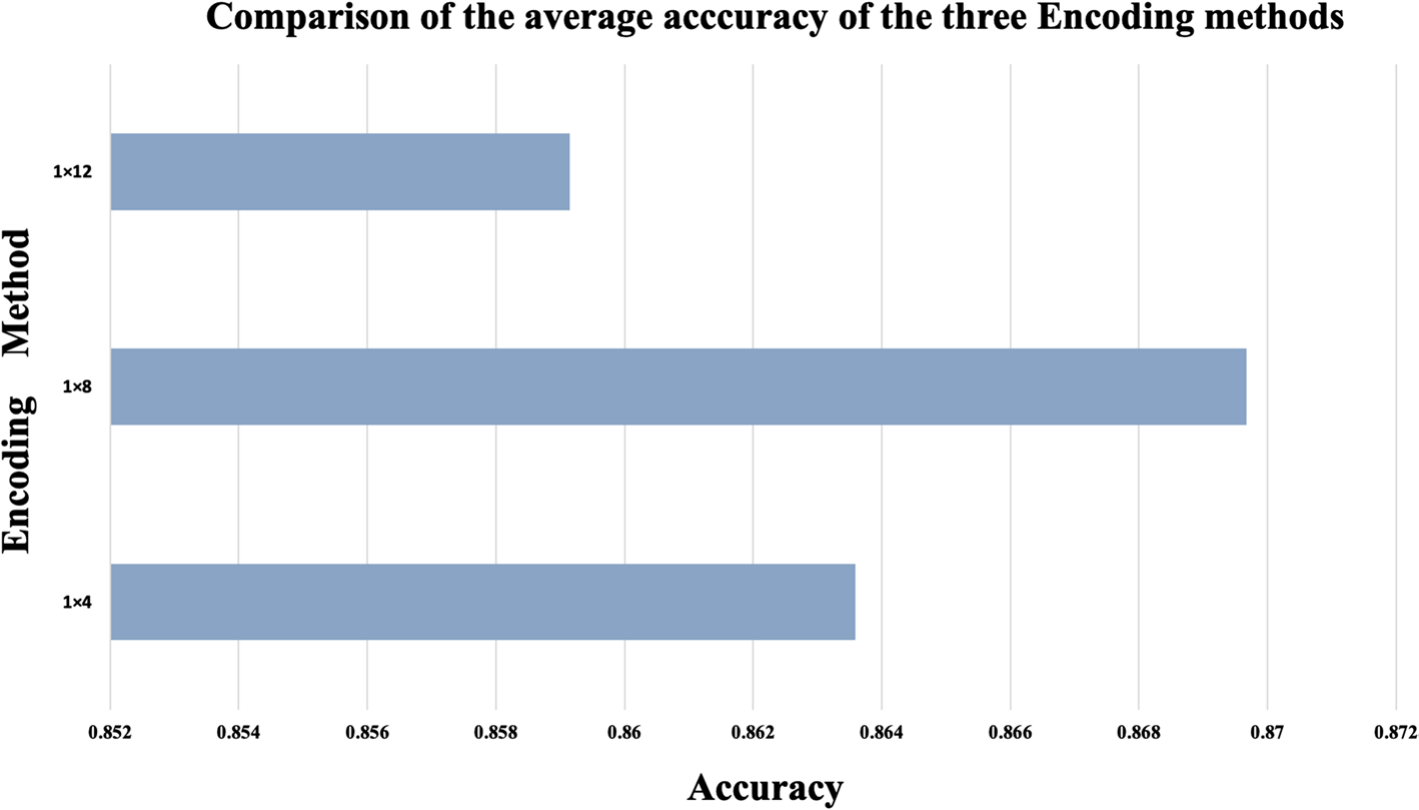
Average cross-validation accuracy of three coding methods with ten folds

The data processed in this paper were all collected from the Rfam database, which included thirteen ncRNAs families (namely, microRNAs, 5S_rRNA, 5.8S_rRNA, ribozymes, CD-box, HACA-box, scaRNA, tRNA, Intron_gpI, Intron_gpII, IRES, leader and riboswitch), with a total of 6320 pieces of non-redundant ncRNAs data, including 320 pieces of IRES data and 500 of each of the remaining ncRNAs families. ncDENSE used a ten-fold cross-validation method in the model training process, and divided ncRNAs data of each family into ten parts, with nine of them as the training set while the remaining one as the test set in turn. Finally, the results of ten cross-validations were averaged. To input ncRNAs sequences into the ensemble deep learning model, one-hot coding was used to encode the bases of each ncRNA as 1*4, 1*8, and 1*12 data in this paper [31]. A (adenine), U (uracil), G (guanine), and C (cytosine) represent the bases of four common ncRNAs, whose coding rules are shown in Table 2, with “N” representing some rare bases. Figure 10 displays the accuracy of the optimal model trained by each fold of the 10-fold cross-validation on the test set by different coding approaches. Figure 11 shows the average accuracy of the three coding methods in the ten-fold cross-validation. According to the results, the 1*8 coding method was more effective than the 1*4 and 1*12 coding methods, as a result, the 1*8 one-hot coding method was used in ncDENSE to digitally code the bases of ncRNAs sequences, and the length of each ncRNAs sequence after coding was L*8 (L is the base number in ncRNAs sequences).

**Table 2:** Three ways of coding bases

Base	A	U	C	G	N
1*12	100000100100	001010000001	000101001000	010000010010	000000000000
1*8	10000010	00101000	00010100	01000001	00000000
1*4	1000	0010	0001	0100	0000

### Methods

**Fig. 12:**
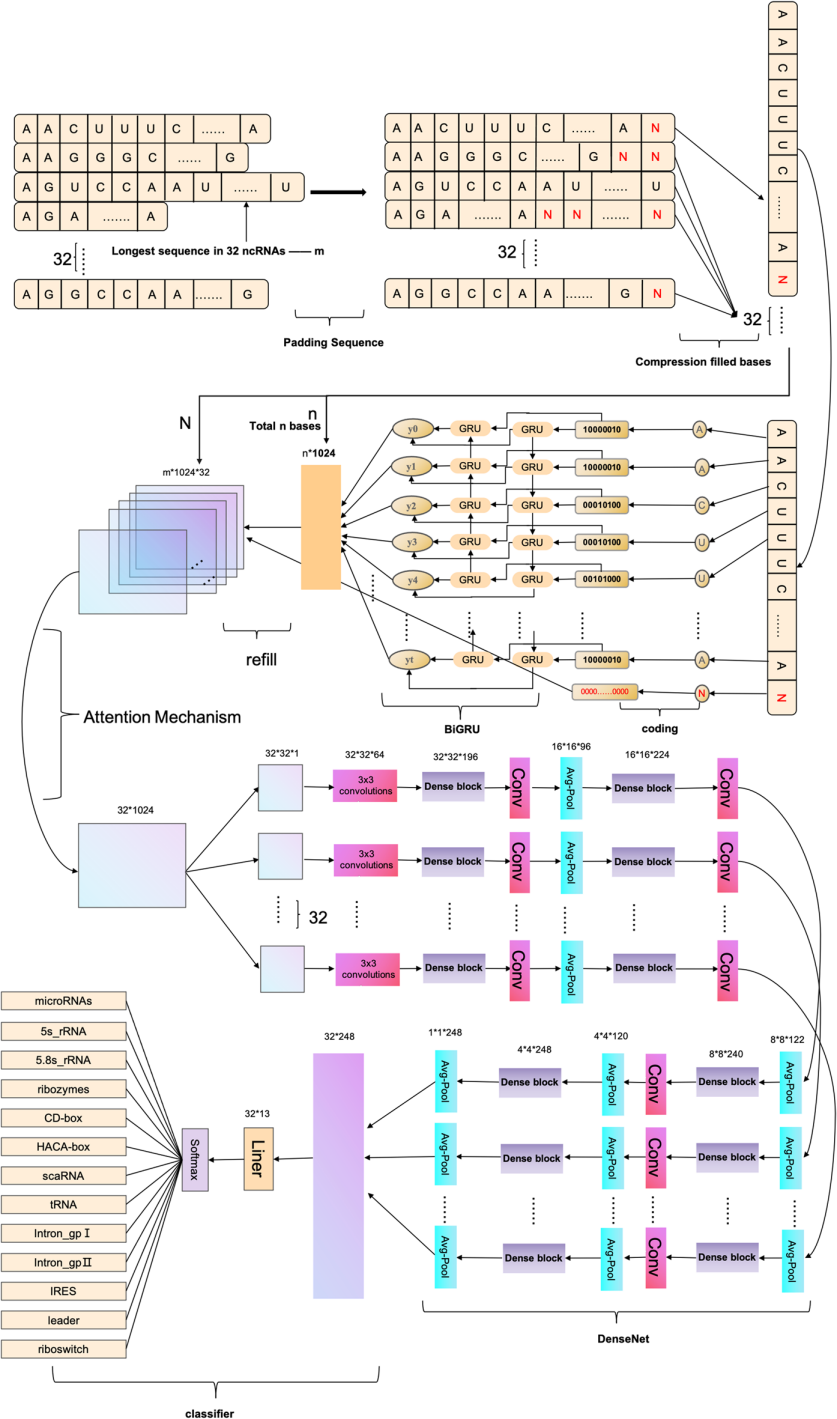
ncDENSE main working process
  2. Calculation of update gate (pink part in Fig. 13 represents the update door) The update gate is used to control the degree to which the state information from the previous moment is brought into the current state. A closer update gate value to 1 indicates that more data are left for the new input, while a value closer to 0 suggests that more new input is forgotten. The update gate value can be calculated by Eq. 7.
    7 $$\begin{aligned}&Z_{t}=\sigma \left( W_{i z} \cdot X_{t}+b_{i z}+W_{h z} \cdot h_{t-1}+b_{h z}\right) \end{aligned}$$
    Where $W_{i z}$ and $W_{h z}$ are the weight matrices of the update gate, $b_{i z}$ and $b_{hz}$ are the deviations of the update gate, separately.
  3. Reset of the current memory content
    8 $$\begin{aligned}{\hat{h}}_{t}=\tanh \left( {W_{it}} \cdot {X_{t}}+{b_{it}}+{r_{t}} \cdot \left( {W_{ht}} {h_{t-1}}+b_{ht}\right) \right) \end{aligned}$$
    Where $W_{it}$ and $W_{ht}$ represent the weight matrices, $b_{it}$ and $b_{ht}$ are the deviations, and ${\hat{h}}$ represents the information after the current moment of calculation.
  4. GRU output calculation
    9 $$\begin{aligned}&h_{t}=\left( 1-Z_{t}\right) \cdot {{\hat{h}}}_{t-1}+Z_{t} \cdot {\hat{h}}_{t} \end{aligned}$$
    Where $h_{t}$ indicates forgetting some information in ${{\hat{h}}}_{t-1}$ passing down from the previous moment and adding some information in ${\hat{h}}_{t}$ of the current moment to form the final memory passed to the next moment.

The ncDENSE method utilized an ensemble deep learning model consisting of three components, namely, dynamic Bi-GRU, Attention Mechanism, and DenseNet. Dynamic Bi-GRU is mainly responsible for capturing the long-term dependencies of ncRNAs sequences to extract their contextual feature information. Meanwhile, Attention Mechanism mainly functions to assign different weights to the features extracted by Bi-GRU according to their importance. While DenseNet extracts local features of ncRNAs sequences by dense concatenation. At last, the ncRNAs sequence features extracted by dynamic Bi-GRU, Attention Mechanism, and DenseNet were input to the full-connected layer for ncRNAs family classification. The ncDENSE method proposed in this paper set the number of ncRNAs sequences in each batch to 32. The main working principle of the ncDENSE method is presented in Fig. 12.

1. Dynamic Bi-GRU
  Dynamic Bi-GRU abandons the traditional padding and cutting of ncRNAs sequences, which inputs all base information in ncRNAs sequences of different lengths into Bi-GRU to avoid the loss of base information or the increase in useless information in ncRNAs sequences. Therefore, it prevents the Bi-GRU model from extracting the redundant feature information of ncRNAs sequences or losing the important feature information of ncRNAs sequences. The ncRNAs sequences are data with closely linked contextual information. The bidirectional RNN model can be well applied in extracting the contextual features of ncRNAs sequences by gating information not only to retain the base information before each base but also to record the base information after the current base. The working principle of dynamic Bi-GRU is shown in Fig. 12. GRU is a variant of LSTM, which is simpler than the structure of LSTM and can effectively solve the problem of long dependency in RNN networks. The GRU unit has only two gate information, including update gate and reset gate, which can improve the model’s computing efficiency. The GRU unit structure is schematically shown in Fig. 13, and the operating working principle can be divided into the following four parts.
  1. Calculation of reset gate (green part in Fig. 13 represents the reset door)
    The reset gate determines how the new input is combined with the previous information. A larger value of the reset gate represents that it is necessary to remember more information from the previous moment, and that more new input ($X_{t}$) is combined with the previous memory ($h_{t-1}$). Conversely, a smaller value of the reset gate indicates that less information should be remembered from the previous moment and less new input ($X_{t}$) should be combined with the previous memory ($h_{t-1}$). The rest gate value can be calculated by Eq. 6.
    6 $$\begin{aligned}&r_{t}=\sigma \left( W_{i r} \cdot X_{t}+b_{i r}+W_{h r} \cdot h_{t-1}+b_{h r}\right) \end{aligned}$$
    Where $W_{i r}$ and $W_{h r}$ are the weight matrices of the reset gate, $b_{i r}$ and $b_{h r}$ represent the deviations of the reset gate, $X_{t}$ is the information input at moment t, $h_{t-1}$ stands for the information passed in at moment t-1 to moment t, and $\sigma$ means the activation function sigmoid.

**Fig. 13:**
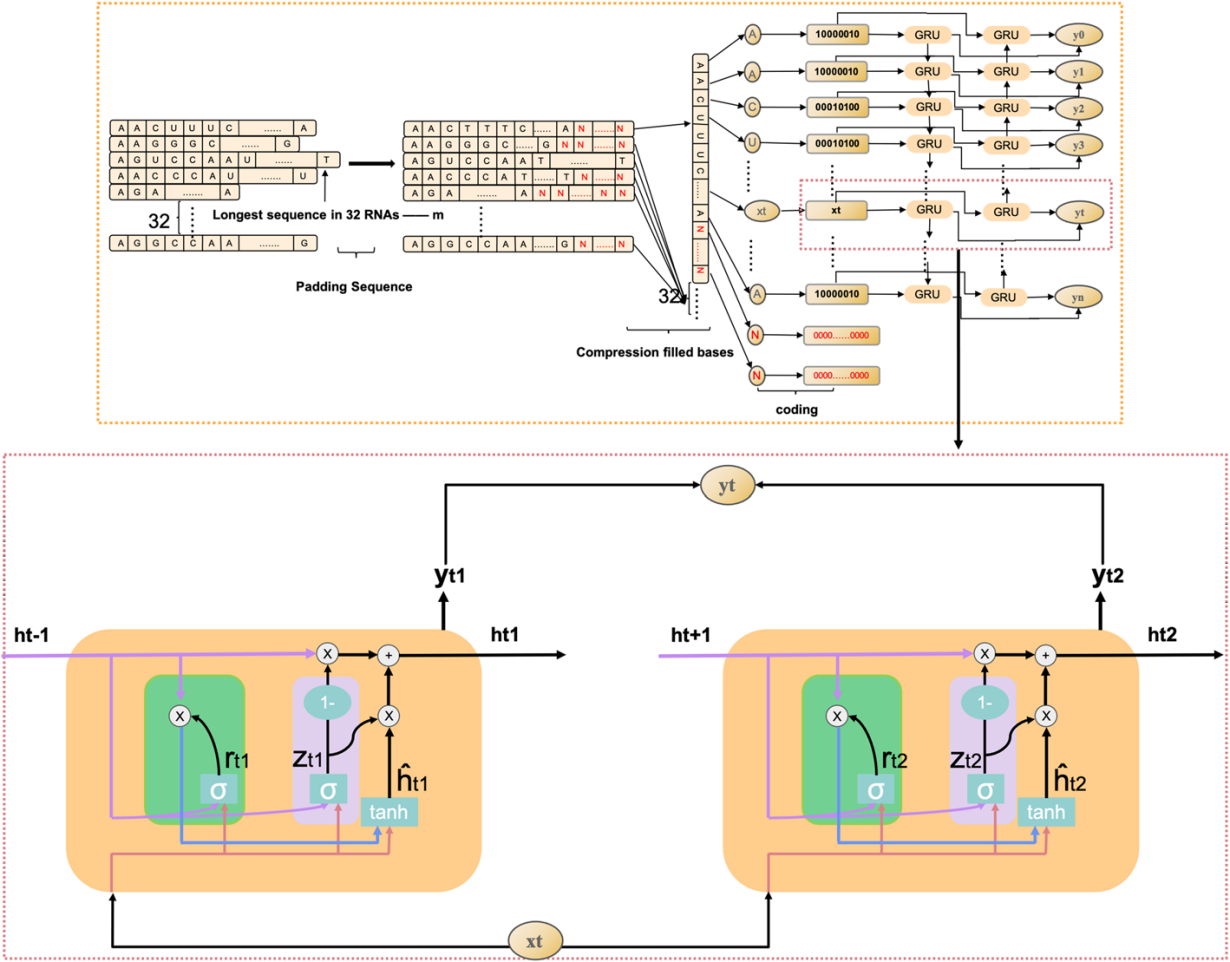
Dynamic Bi-GRU working principle
2. Attention Mechanism
  The core idea of the attention mechanism is to assign more weights to important input information, so that attention can be subtly and rationally shifted to ignore irrelevant information and amplify important information. In this way, the sensitivity of information reception and processing speed in the focused attention area are greatly improved. The attention mechanism works as shown in Eq. 10.
  10 $$\begin{aligned}&C=\sum _{j=1}^{L x} a_{j} h_{j} \end{aligned}$$
  Where C is the output of the attention mechanism, Lx indicates the length of the input RNA sequence, a suggests the coefficient assigned to the jth hidden state, and h represents the jth hidden state. The attention mechanism is characterized by its ability to well accomplish the task of focusing ncDENSE on the important fragments of the ncRNA family.
3. DenseNet
  The design of DenseNet is inspired by ResNet. A dense connection mechanism is proposed in DenseNet by improving the connection of ResNet. The main idea of the dense connection mechanism is to connect all layers to each other, and each layer receives information from all previous layers as input. In this paper, a 4-layer Dense block was used, and each Dense block had a total of 10 connections, as shown in Fig. 14. The calculation of each layer is shown in Eq. 11.
  11 $$\begin{aligned}&X_{i}=H \quad \left( \left[ X_{1}, X_{2}, X_{3} \ldots \ldots X_{i-1}\right] \right) \end{aligned}$$
  Where $X_{i}$ is the result of layer i, and H uses the structure of BN+ReLU+3*3CONV in Eq. 11. Each sequence of ncRNAs is extracted by four dense blocks, and the sizes of the four dense block output features are 32*32*196, 16*16*224, 8*8*240, and 4*4*248, respectively. The transition layer consists of the Conv layer and Avg-Pool layer, which can downscale the features output from the dense block, and the dimensions of three transitions after downscaling are 16*16*96, 8*8*122, and 4*4*120 separately. The size of the last layer of Avg-Pool is 1*1*248, and the last 32 ncRNAs sequences are reshaped together as 32*248 for classification by Liner in PyTorch for the full connection layer.

**Fig. 14:**
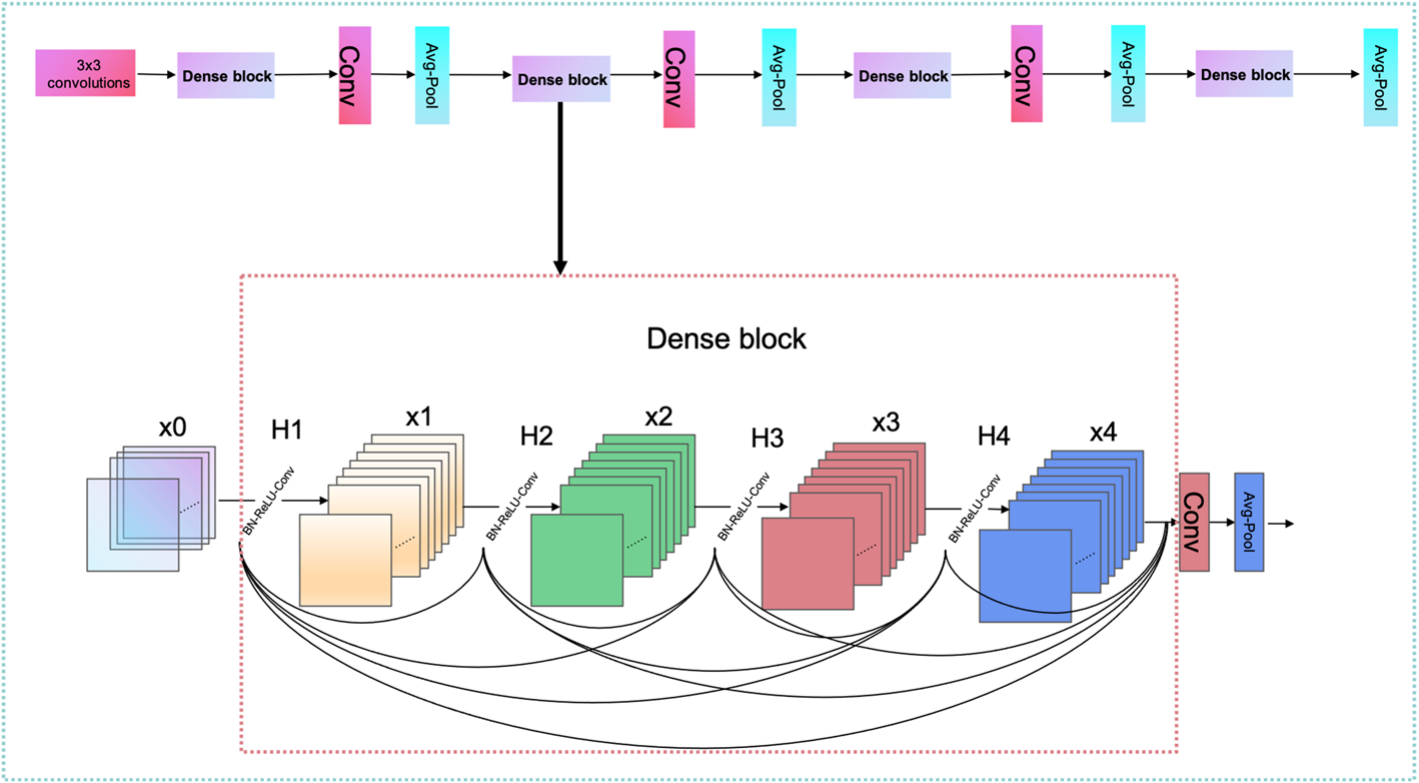
DenseNet’s working principle

## Data Availability

All the original experimental data can be available from the citations, and the ncDENSE method can be available at https://github.com/ck-fighting/ncDENSE
